# MGMT inhibition in ER positive breast cancer leads to CDC2, TOP2A, AURKB, CDC20, KIF20A, Cyclin A2, Cyclin B2, Cyclin D1, ERα and Survivin inhibition and enhances response to temozolomide

**DOI:** 10.18632/oncotarget.25696

**Published:** 2018-07-03

**Authors:** George C. Bobustuc, Amin B. Kassam, Richard A. Rovin, Sheila Jeudy, Joshua S. Smith, Beth Isley, Maharaj Singh, Nathan David Simmons, Kalkunte S. Srivenugopal, Santhi D. Konduri

**Affiliations:** ^1^ Aurora Research Institute, Milwaukee, WI, USA; ^2^ Aurora Neurosciences Innovation Institute, Milwaukee, WI, USA; ^3^ Florida Hospital, Orlando, FL, USA; ^4^ Texas Tech University Health Sciences Center, Amarillo, TX, USA

**Keywords:** MGMT, BG, temozolomide, ER-positive breast cancer

## Abstract

The DNA damage repair enzyme, O^6^-methylguanine DNA methyltransferase (MGMT) is overexpressed in breast cancer, correlating directly with estrogen receptor (ER) expression and function. In ER negative breast cancer the MGMT promoter is frequently methylated. In ER positive breast cancer MGMT is upregulated and modulates ER function. Here, we evaluate MGMT’s role in control of other clinically relevant targets involved in cell cycle regulation during breast cancer oncogenesis. We show that O^6^-benzylguanine (BG), an MGMT inhibitor decreases CDC2, CDC20, TOP2A, AURKB, KIF20A, cyclin B2, A2, D1, ERα and survivin and induces c-PARP and p21 and sensitizes ER positive breast cancer to temozolomide (TMZ). Further, siRNA inhibition of MGMT inhibits CDC2, TOP2A, AURKB, KIF20A, Cyclin B2, A2 and survivin and induces p21. Combination of BG+TMZ decreases CDC2, CDC20, TOP2A, AURKB, KIF20A, Cyclin A2, B2, D1, ERα and survivin. Temozolomide alone inhibits MGMT expression in a dose and time dependent manner and increases p21 and cytochrome c. Temozolomide inhibits transcription of TOP2A, AURKB, KIF20A and does not have any effect on CDC2 and CDC20 and induces p21. BG+/-TMZ inhibits breast cancer growth. In our orthotopic ER positive breast cancer xenografts, BG+/-TMZ decreases ki-67, CDC2, CDC20, TOP2A, AURKB and induces p21 expression. In the same model, BG+TMZ combination inhibits breast tumor growth *in vivo* compared to single agent (TMZ or BG) or control. Our results show that MGMT inhibition is relevant for inhibition of multiple downstream targets involved in tumorigenesis. We also show that MGMT inhibition increases ER positive breast cancer sensitivity to alkylator based chemotherapy.

## INTRODUCTION

MGMT, a DNA repair protein, mediates resistance to alkylating agents. Increased MGMT expression level is exclusively a tumor phenotype trait as MGMT levels in normal tissues do not vary between patients with benign or malignant disease [1]. In breast cancer, MGMT and ER are largely expressed along a quantitative and qualitative continuum. In ER negative breast cancer, the MGMT promoter is frequently methylated in a BRCA1 dependent manner, with highest rate of MGMT promoter methylation (>60%) in wild-type BRCA1 triple negative breast cancer [2]. In ER positive breast cancer MGMT levels are up to 4-fold higher than in the normal breast [1, 3]. MGMT expression increases during tumor progression and correlates with the overall metastatic tumor burden and with the organ/tissue type harboring the metastatic disease [1, 4]. Furthermore MGMT expression level correlates with ERα functional status / phosphorylation phenotype, as, when MGMT levels are 3-4 fold higher than in the normal breast tissue, ERα phosphorylation pattern changes from a tamoxifen sensitive (high p-ERα Ser167/low p-ERα Ser118) to a tamoxifen resistant (low p-ERα Ser167/high p-ERα Ser118 ERα) phosphorylation phenotype [4–7].

MGMT has been reported to play a role in integrating DNA damage/repair related signals with replication and cell cycle progression [8, 9]. In this study we evaluate the potential MGMT’s role in control of therapeutic, clinically relevant, cell cycle regulators involved in breast cancer oncogenesis (CDC2, TOP2A, AURKB, CDC20, KIF20A, Cyclin A2, Cyclin B2, Cyclin D1).

O^6^ benzylguanine (BG) inhibits MGMT and potentiates the cytotoxicity of alkylating agents [10]. BG is a pseudosubstrate which reacts directly with both cytoplasmic and nuclear MGMT resulting in the covalent transfer of a benzyl group to the active cysteine site, leading to MGMT protein degradation. Depletion of MGMT with BG has been shown to enhance activity of alkylating agents [10–12].

Temozolomide is a monofunctional imidazotetra-zinone alkylator approved for the treatment of high-grade glioma [13–18]. Temozolomide based trials for phenotype non-specific metastatic breast cancer have not shown any survival benefit [19, 20], underscoring the significantly higher prevalence (70%) of the ER positive breast cancer, associated with high MGMT expression levels. When Temozolomide was used in combination with Xeloda for intraparenchymal brain metastatic disease due to breast cancer in a phase 1 study [21] the combination was found to be active, likely due to an inherent, phenotype based, two fold, selection sample bias, the selection of a ER negative phenotype (targeting the brain and spinal cord parenchyma as opposed to ER positive breast cancer which preponderantly involves the skull and dura and spares the brain and spinal cord parenchyma) and an overrepresentation of the triple negative subset (where MGMT promoter methylation is frequent) in the brain metastatic disease breast cancer patients. Results of this small study hint at the importance of MGMT status when targeting breast cancer with alkylator based combinations.

CDC2 (Cell Division Cycle protein 2) plays a role in the control of G2/M transition. MGMT overexpressing cells show a transient cell cycle G2/M arrest [9]. CDC2 over expression is found in high grade breast cancer and is associated with a poor outcome [22].

TOP2A (Topoisomerase 2-alpha), a nuclear enzyme involved in chromosome condensation, chromatid separation and relief of torsional stress during DNA transcription and replication [23], is a predictive marker of anthracycline efficacy. TOP2A amplification is frequent in breast cancer correlating with larger, higher grade tumors and positive lymph nodes [24]. High TOP2A amplification, especially in patients with concomitant high Her2neu amplification, is predictive of high pathologic complete response rates (> 50%) when treated with anthracycline based regimens [25, 26]. In ER positive, Her2neu negative breast cancer, TOP2A over expression correlates with an increased risk of relapse in patients with an intermediate RS (recurrence score) as determined by Oncotype DX [27].

AURKB (Aurora Kinase B) is a mitotic checkpoint kinase. ARUKB overexpression leads to p53 functional inactivation [28], and is found in a variety of cancers including breast cancer, multiple myeloma, colorectal, pancreatic and prostate cancers and glioblastomas [29, 30]. In ER positive breast cancer antiestrogen therapy resistance has been linked to overexpression of ARUKB [31].

CDC20 (Cell Division Cycle protein 20) is associated with extremely poor outcome in breast cancer [32].

KIF20A, a microtubulin associated kinesin coordinating intracellular transport in cell division, is overexpressed in a variety of cancers and is associated with Taxol resistance [33].

Cyclin A2 overexpression is common in breast cancer and is associated with high propensity for epithelial-mesenchymal transition (EMT) and metastasis [34, 35].

Cyclin B2, another regulator of cell division cycle, is commonly overexpressed in breast cancer and is associated with increased invasion and metastasis [36].

Cyclin D1, another regulator of cell division, forms the Cyclin D1-CDK4/CDK6 complex, a promising target in the treatment of adjuvant/post-neoadjuvant ER positive breast cancer [37].

Survivin, an antiapoptotic gene, specifically upregulated during the G2/M transition [38], is overexpressed in human cancers and is involved in chemoresistance. Survivin has been reported to be under the control of MGMT in a pancreatic cancer model [39]. Survivin over expression in breast cancer has been correlated to both tumor progression and a poor prognosis [40].

This study is aimed to evaluate expression and transcription correlations between MGMT and already clinically relevant cell cycle regulators involved in breast cancer oncogenesis, chemotherapy resistance (TOP2A for anthracyclines, KIF20A for taxol) [25, 26, 33] and hormonal therapy resistance (ARUKB) [31]. This study also aims to show that, in ER positive breast cancer, usually associated with high MGMT levels, alkylator (temozolomide) mediated cytotoxic effect is restored by MGMT inhibition.

## RESULTS

### Correlative expression of ERα and MGMT in breast cancer cells

We investigated ERα and MGMT expression in breast cancer cells - ER positive cells: MCF7 (MGMT +), T-47-D (MGMT +), ZR-75-1 (MGMT +); ER negative cells: ZR-75-30 (ER weakly positive and MGMT weakly positive), HCC 1428 (ER weakly positive and MGMT weakly positive), MDA MB 468 (ER negative and MGMT positive). MGMT expression was higher in ZR-75-1 followed by MCF7, HCC 1428, MDA MB 468, T-47-D with low expression in ZR-75-30 cells. ERα expression was higher in ZR-75-1 and MCF-7 followed by T-47-D, with low expression in ZR-75-30, HCC 1428 and MDA MB 468 cells were negative (Supplementary Figure 1).

### BG induced MGMT inhibition leads to inhibition of CDC2, TOP2A, AURKB, KIF20A, CDC20 expression

**Figure 1:**
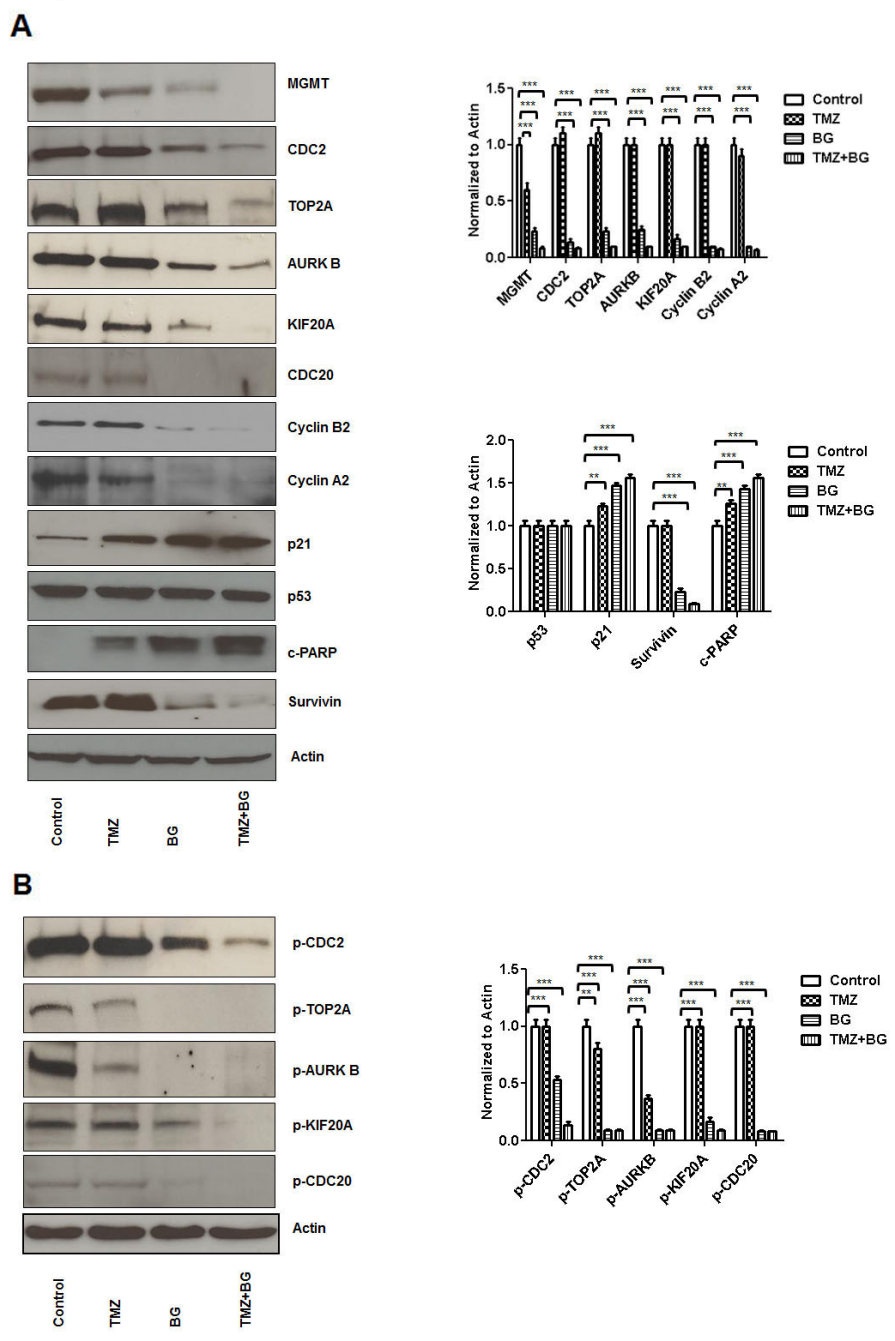
BG induced MGMT inhibition leads to inhibition of CDC2, TOP2A, AURKB, KIF20A, CDC20 expression MCF7 cells were treated with either BG or TMZ alone or combination (TMZ+BG). BG induced MGMT inhibition correlated to decreased expression of CDC2, TOP2A, AURKB, KIF20A, CDC20, cyclin B2 and A2 and survivin **(A)**. Temozolomide had a limited inhibitory effect on MGMT expression and did not influence expression of CDC2, TOP2A, AURKB, KIF20A, CDC20, Cyclin A2 and Cyclin B2 and survivin while it mildly induced c-PARP and p21 (A). When compared to BG alone, the BG+TMZ combination therapy further decreased expression of MGMT, CDC2, TOP2A, KIF20A, Cyclin A2, and Cyclin B2 (A). Expression of phosphorylated fractions followed largely a similar pattern, where BG induced MGMT inhibition correlated with significant reduction in the p-CDC2, p-TOP2A, p-ARUKB, p-KIF20A and p-CDC20 **(B)**.

In our model (MCF7 cells) a BG induced MGMT expression inhibition of 70% correlated to decreased expression of CDC2 (by 80%), TOP2A (by 60%), AURKB (by 60%), KIF20A (by 70%) and CDC20 (by 100%) (Figure 1A). Temozolomide alone decreased MGMT expression by 30% but did not influence expression of CDC2, CDC20, TOP2A, AURKB, KIF20A, Cyclin A2 and Cyclin B2 and survivin while it induced c-PARP and p21(by 30%) (Figure 1A). When compared to BG alone, the BG+TMZ combination therapy further decreased expression of MGMT (by 55%), CDC2 (by 23%), TOP2A (by 60%), KIF20A (by 20%), Cyclin A2 (by 15%) and Cyclin B2 (by 10%) (Figure 1A). Expression of phosphorylated correlates followed a largely similar pattern where BG led to 50% reduction in the p-CDC2, 85% reduction in p-TOP2A, p-ARUKB and p-CDC20 and 70% reduction in p-KIF20A (Figure 1B).

### BG induced MGMT inhibition leads to inhibition of Cyclin D1 and AURKB expression independent of presence or absence of 17-β estradiol (E2)

BG and BG+TMZ (MCF-7 and ZR-75-1 cells) significantly decreased correlative protein expressions (MGMT, ERα, Cyclin D1 and Aurora Kinase B) both in presence and absence of 17 – β estradiol (Supplementary Figure 4).

### siRNA MGMT inhibition leads to decreased CDC2, TOP2A, ARUKB, KIF20A, Cyclin B2, Cyclin A2 and Survivin expression

**Figure 2:**
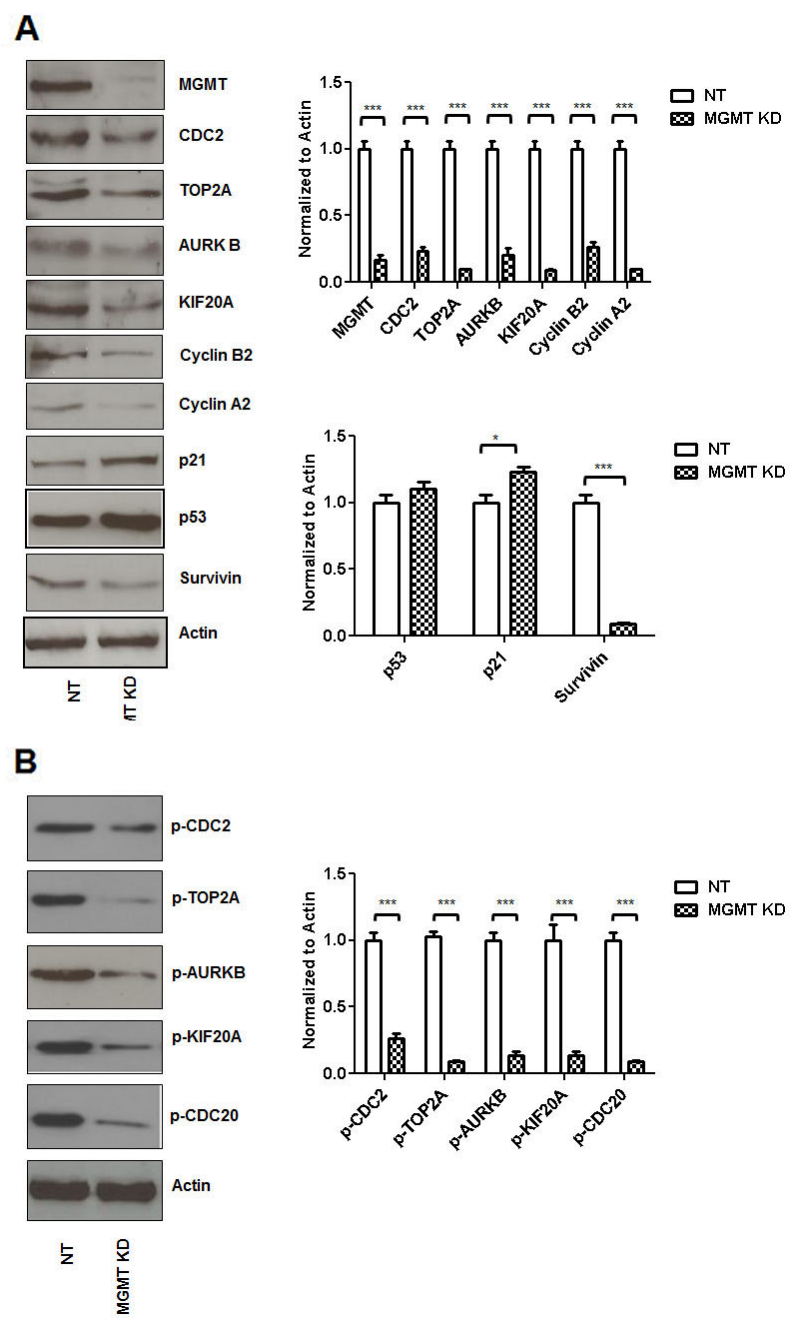
siRNA MGMT inhibition leads to decreased CDC2, TOP2A, ARUKB, KIF20A, Cyclin B2, Cyclin A2 and Survivin expression siRNA MGMT inhibition (MCF7 cells) correlated with a decrease in expression of CDC2, TOP2A, AURKB, KIF20A, Cyclin B2, Cyclin A2 and Survivin **(A)**. When MGMT was silenced p53 expression was slightly increased and this correlated with mild induction of p21 expression. siRNA MGMT inhibition also correlated with decreased p-CDC2, p-TOP2A, p-AURKB, p-KIF20A, p-CDC20 **(B)**.

An 80% siRNA inhibition of MGMT expression (MCF7 cells) correlated with a decrease in expression of CDC2 (by 70%), TOP2A (by 90%), AURKB (by 75%), KIF20A (by 85%), Cyclin B2 (by 70%), Cyclin A2 (by 85%) and Survivin (by 90%) (Figure 2A). When MGMT was silenced p53 expression was slightly increased in these cells and this correlated with induction of p21 (20%) expression in our system. Eighty percent MGMT inhibition by siRNA drastically decreased p-CDC2 (by 70%), p-TOP2A (by 90%), p-AURKB (by 90%), p-KIF20A (by 80%), p-CDC20 (by 85%) (Figure 2B).

### BG+/-TMZ therapy decrease CDC2, TOP2A, AURKB, CDC20, KIF20A transcription and induce p21 transcription

**Figure 3:**
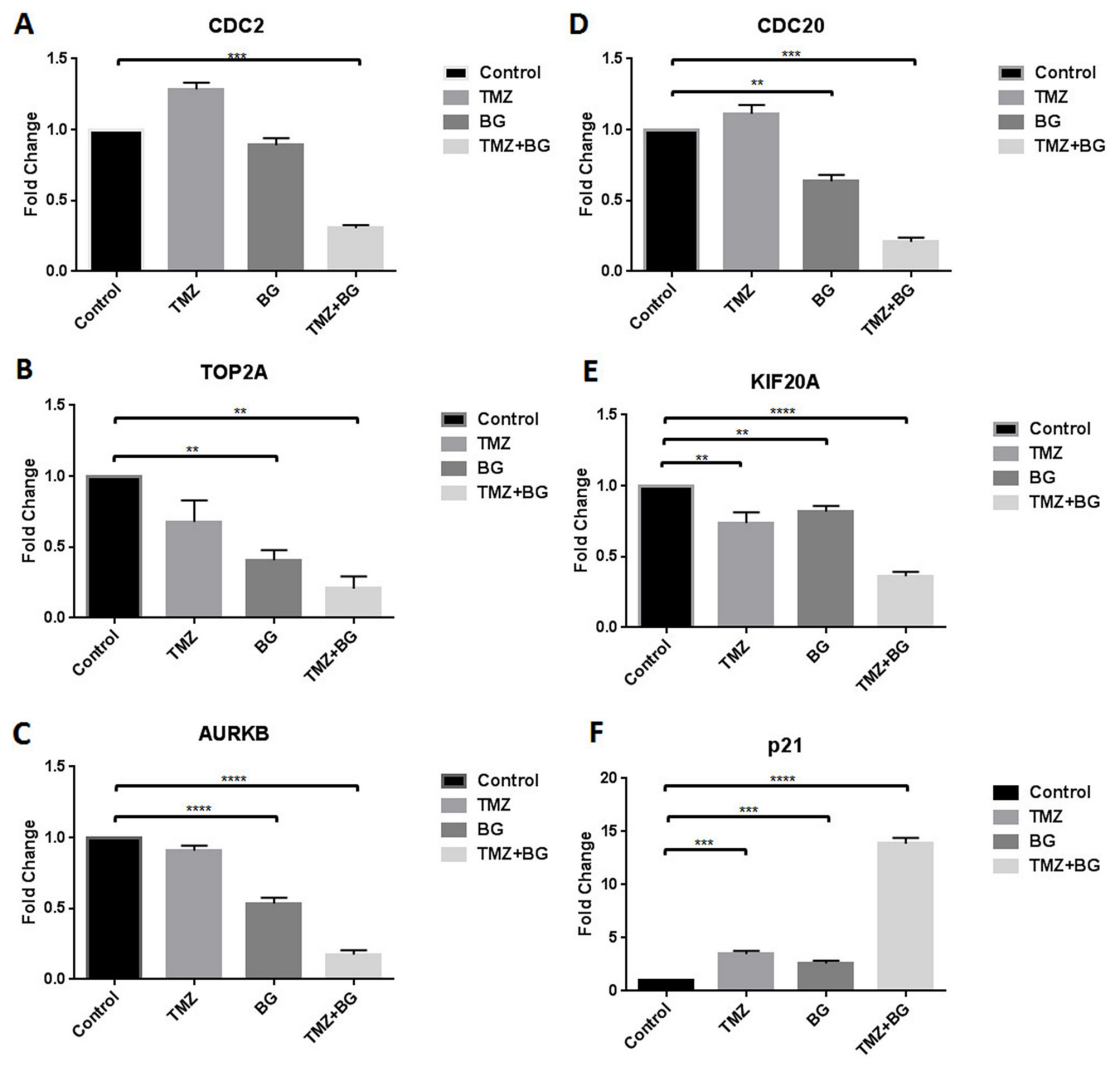
BG+/-TMZ therapy decrease CDC2, CDC20, TOP2A, KIF20A, AURKB transcription and induce p21 transcription MCF7 cells were treated with BG or TMZ or BG+TMZ combination. BG alone decreased transcription of CDC2, TOP2A, AURKB, CDC20 and KIF20A **(A-E)**. TMZ alone decreased transcription of TOP2A, AURKB and KIF20A (B, C, E). Combination therapy further decreased transcription of CDC2, TOP2A, AURKB, CDC20 and KIF20A (A-E). Transcription of p21 was increased by TMZ, BG and combination therapy **(F)**.

BG alone decreased transcription (MCF7 cells) of (A) CDC2 (by 3.3 fold), (B) TOP2A (by 1.6 fold), (C) AURKB (by 2 fold), (D) CDC20 (by 2.5 fold) and (E) KIF20A (by 5 fold) (Figure 3A–3E). TMZ alone decreased transcription of TOP2A (2.5 fold); AURKB (by 2.2 fold) and KIF20A (by 3.3 fold) (Figure 3A–3E). Combination therapy further decreased transcription of all targets studied. Transcription of p21 was increased by TMZ (2.5 fold), BG (2 fold) and combination therapy (14 fold) (Figure 3F).

### TMZ inhibits MGMT expression and induces p21 and cytochrome C expression in breast cancer cells

**Figure 4:**
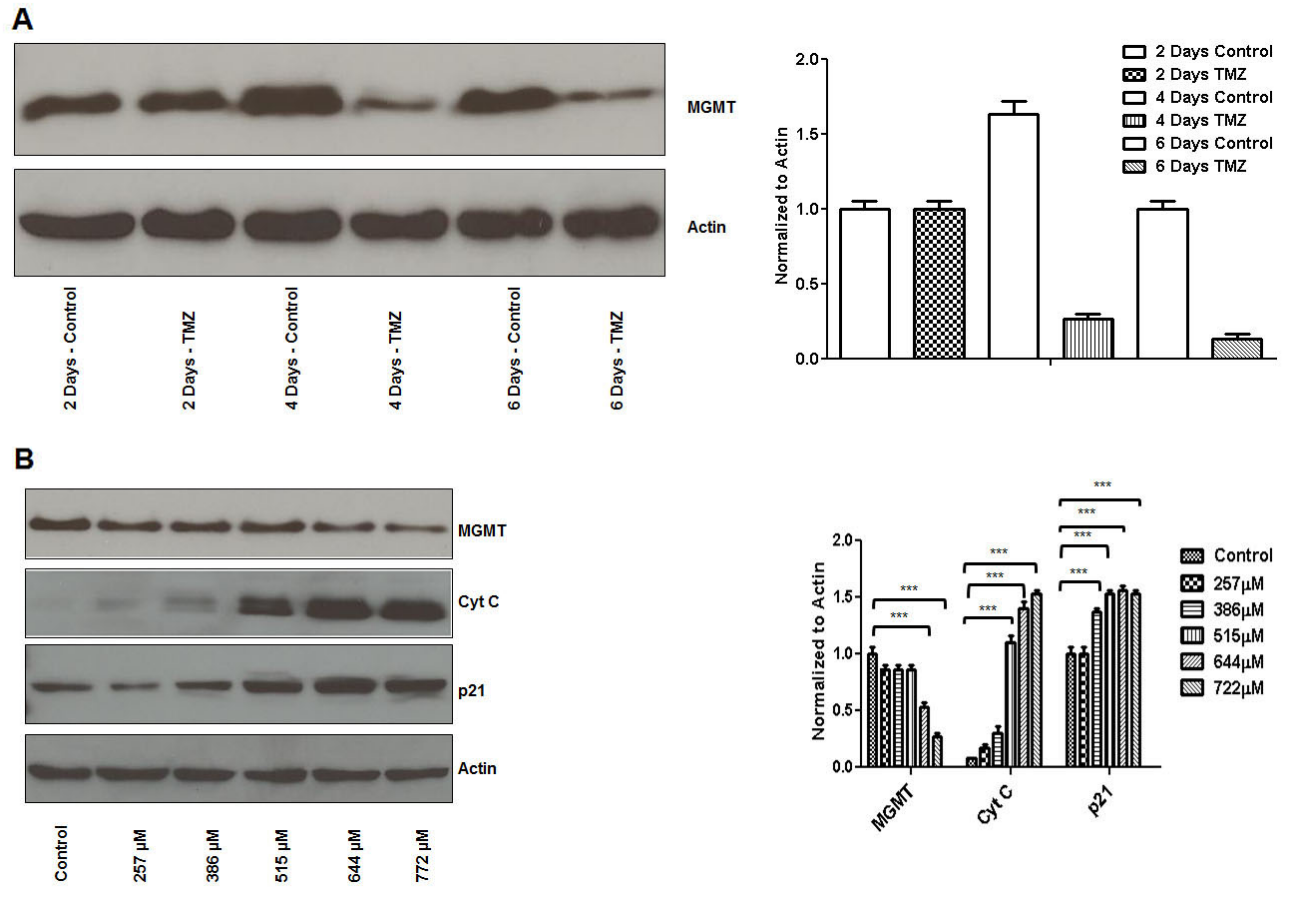
TMZ inhibits MGMT expression and induces p21 and cytochrome C expression in breast cancer cells MCF7 cells were plated overnight and subsequently treated with TMZ (644μM) on an every other day basis with harvest time points at 2, 4 and 6 days. TMZ induced MGMT expression inhibition was significant at 4 days **(A)**. MCF7 cells were treated with different concentrations of TMZ (257μM to 772μM) on an every other day basis before harvest at 4 days. TMZ induced MGMT inhibitory effect correlated with increased cyt C and p21 expression **(B)**.

TMZ induced MGMT expression inhibition (MCF7 cells) was significant at 4 days in our system (∼80%) (Figure 4A). TMZ induced MGMT inhibitory effect correlated with increased expression of cyt C and p21 expression (Figure 4B).

### BG decreases MGMT activity and sensitizes breast cancer cells to TMZ

**Figure 5:**
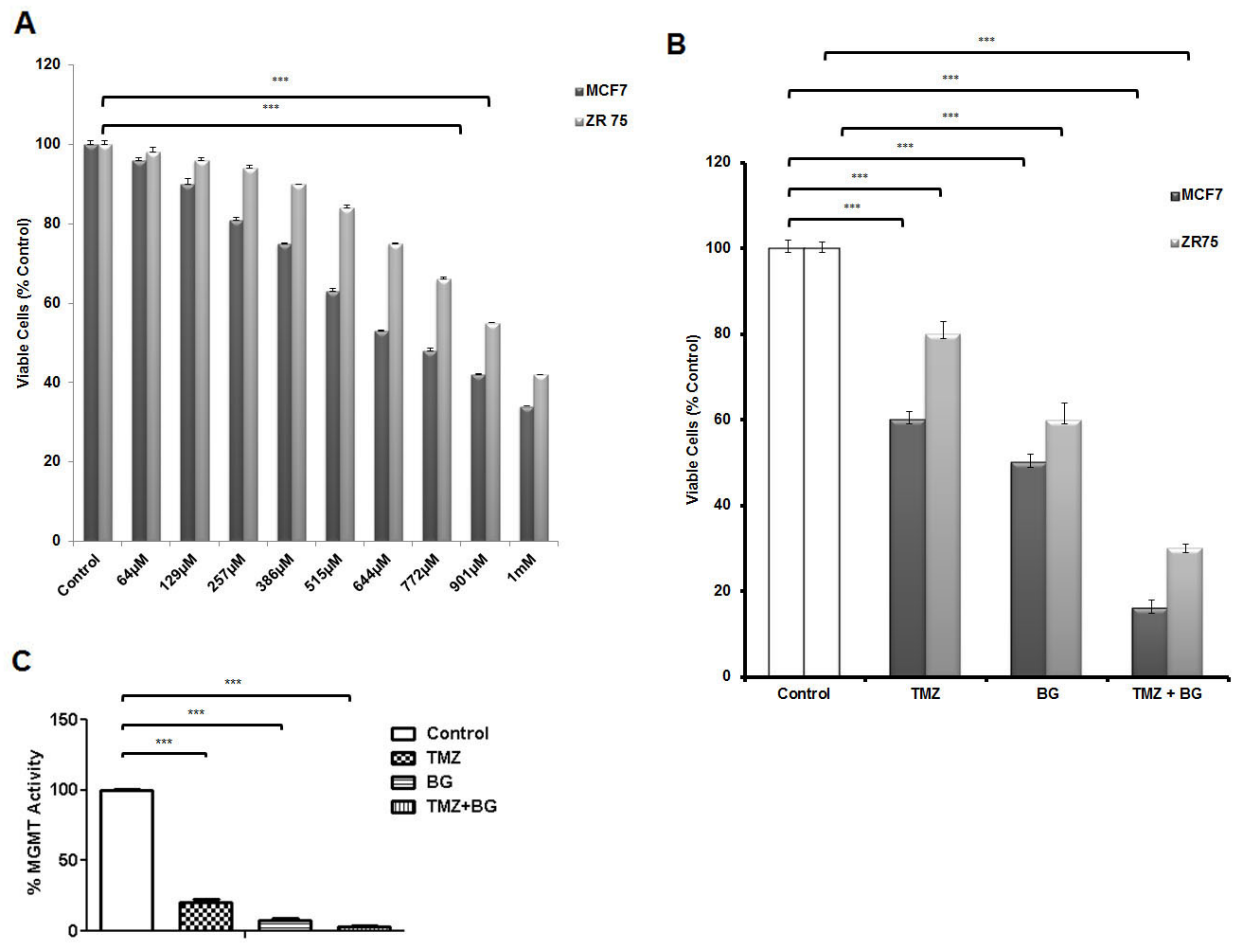
BG decreases MGMT activity and sensitizes breast cancer cells to TMZ Human breast cancer cells MCF7 and ZR-75 were treated with different concentrations of TMZ (scaling from 64μM to 1mM) every other day before harvest at 4 days. TMZ inhibits breast cancer cell growth in a dose dependent manner with TMZ IC50 for MCF7 ∼ 644μM and TMZ IC50 for ZR-75 ∼ 900μM **(A)**. MCF7 and ZR-75 cells were treated with BG (IC50 140μM) in the presence or absence of TMZ (TMZ IC50 644μM for MCF7 and TMZ IC50 900μM for ZR-75) and retreated 2 days later prior to harvesting the cells at 4 days for ATP assays. BG + TMZ further decreased MCF7 and ZR-75 cell viability when compared to BG alone **(B)**. BG + MGMT combination further decreased MGMT activity when compared to BG or TMZ alone **(C)**.

TMZ inhibits breast cancer cell growth (MCF7 and ZR-75) in a dose dependent manner with TMZ IC50 for MCF7 ∼ 644μM and TMZ IC50 for ZR-75 ∼ 900μM (Figure 5A). Previously, we have reported BG IC50 to be 140 μM in tamoxifen resistant (MCF7) breast cancer cells [4]. TMZ alone decreased MCF7 cell viability by 52% and ZR-75 cell viability by 20%. BG alone decreased MCF7 cell viability by 50% and ZR-75 cell viability by 40%. BG + TMZ further decreased MCF7 cell viability by 80% and ZR-75 cell viability by 70% (Figure 5B). Temozolomide (644 μM) decreased MGMT activity by ∼90% and BG (50 μM) decreased MGMT by ∼95% and the combination decreased MGMT activity by 99% (Figure 5C).

### BG increases (low dose) TMZ inhibitory effect on breast cancer growth irrespective of presence or absence of 17-β estradiol (E2)

Human breast cancer cells MCF7, ZR-75-1, T-47-D, ZR-75-30, HCC -1428 and MDA MB 468 cells were treated with single agents (TMZ 64μM, BG 50μM) or combination therapy (TMZ+BG) and cell viability was measured. Cell viability was significantly decreased by TMZ or BG or TMZ+BG treatment. For MCF-7 viability was decreased by – 54%, 68%, 17%; ZR-75-1 – 81%,97%, 26%; for T-47D viability was decreased by – 86%,54%, 34%; for ZR-75-30 by 67%, 57%,33%; for HCC -1428 – by 74%, 44% and 34% and for MDA MB 468 by – 46%, 118%, 23%. These results show that combination therapy significantly inhibits cell viability compared to single agents and untreated controls (Supplementary Figure 2) Combination therapy significantly decreased cell viability of all cells tested irrespective of presence or absence of 17β - estradiol compared to single agents and untreated controls. In addition, E2 mediated growth was significantly decreased by combination therapy. These results show that combination therapy mediated inhibitory effect is not altered by E2 growth stimulatory effect (Supplementary Figure 3).

### BG increases TMZ inhibitory effect on breast cancer growth *in vivo*

**Figure 6:**
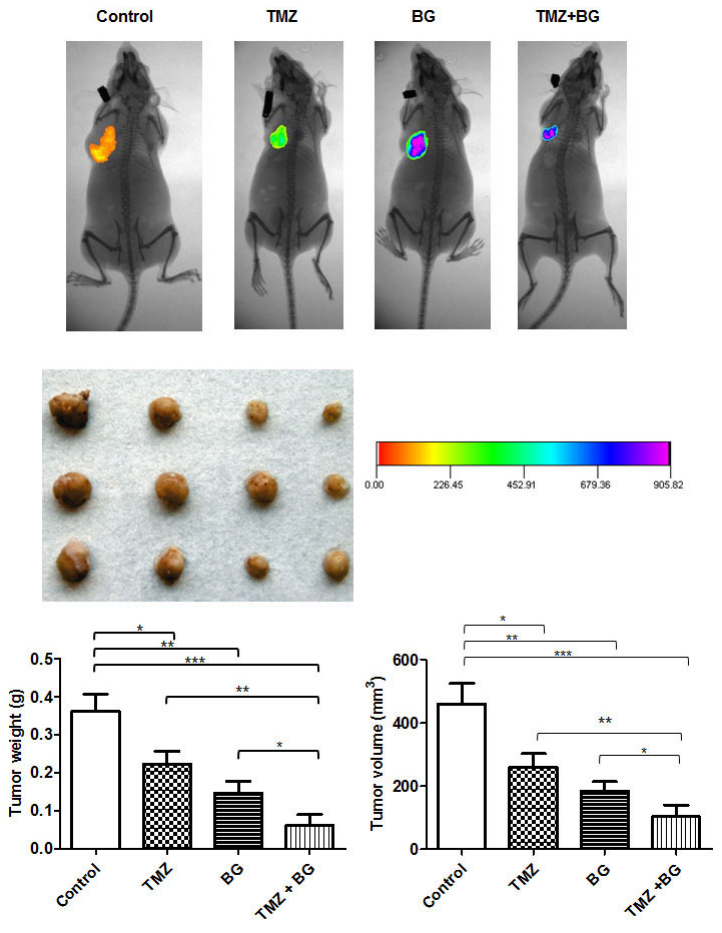
BG increases TMZ inhibitory effect on breast cancer growth *in vivo* The combination BG+TMZ produced the greatest decrease (80%) in median tumor volume (103.5 mm3) as compared with control mice (460.3 mm^3^) (p< 0.0001) (Figure 6). Tumor weight was also significantly reduced (80%) in mice treated with BG + TMZ combination therapy (61.9 mg) as compared with control mice (361.3 mg) (p<0.0003) (Table 1). TMZ alone decreased tumor volumes and weights by 45% and BG alone by 65%. Body weight was not changed among all treatment groups as compared with control mice. No visible liver metastases were present (enumerated with the aid of a dissecting microscope) in any of the treatment groups. All slides were reviewed by a board certified pathologist.

The data summarized in Table 1 shows the daily treatment with single agent BG or BG+TMZ combination significantly decreased median tumor volume and weight as compared to both single agent TMZ treated mice and control mice. The combination BG+TMZ produced the greatest decrease (80%) in median tumor volume (103.5 mm3) as compared with control mice (460.3 mm^3^) (p< 0.0001) (Figure 6). Tumor weight was also significantly reduced (80%) in mice treated with BG+TMZ combination therapy (61.9 mg) as compared with control mice (361.3 mg) (p<0.0003) (Table 1). TMZ alone decreased tumor volumes and weights by 45% and BG alone by 65%.

**Table 1:** Benzylguanine sensitizes breast cancer cells to temozolomide

		Breast Tumors					
		Tumor Volume (mm^3^)		Tumor Weight (mg)		Body Weight (g)	
Treatment Group^†^	Tumor Incidence	Mean	Range	Mean	Range	Mean	Range
Vehicle Control	10/10	460.3	100.1-664.2	361.3	84.3-515.4	23.55	19.15-26.37
TMZ (100mg/kg)	10/10	259.7^α^	100.7-417.3	222.7^‡^	104.2-379.9	21.06	16.86-24.22
BG (10mg/kg)	10/10	184.1^β^	57.09-306.3	147.2^§^	45.9-282.1	22.42	17.39-26.68
TMZ + BG	10/10	103.5^μ^	23.36-278.4	61.9^#^	24.3-144.1	20.39	17.03-23.42

^†^MCF7-luc human breast cancer cells (7 × 10^6^) were injected into the mammary fat pad of female ovarectomized nude mice. After a palpable tumor was formed, four groups of mice were treated: vehicle solution (daily); TMZ (100mg/kg) by i.p injections twice weekly; BG (10mg/kg) by i.p injection daily. All mice were sacrificed on day 56.

^‡^*P* < 0.0315 compared to control. ^§^*P* < 0.0022 compared to control. ^#^*P* < 0.0003 compared to control. ^α^*P* < 0.0217 compared to control. ^β^*P* < 0.0024 compared to control. ^μ^*P* < 0.0004 compared to control.

### *In vivo* BG induced MGMT inhibition leads to increase in p21 and decrease in Ki-67 expression

**Figure 7:**
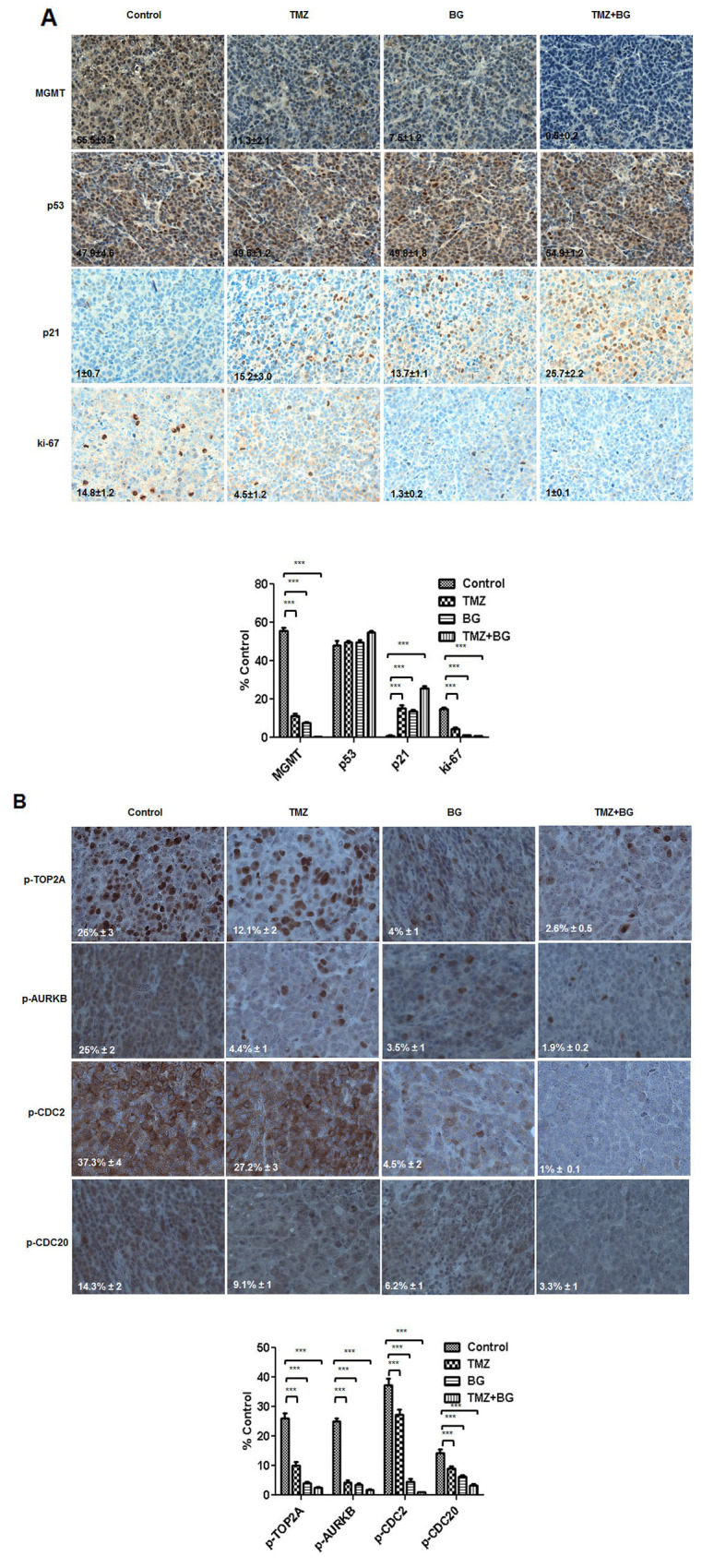
**(A and B)** BG+/-TMZ therapy decrease MGMT, ki-67 and p-TOP2A, p-AURKB, p-CDC2, p-CDC20 and induce p21 in breast tumors derived from mice. Representative samples (40X) are shown. IHC correlative quantified expression analysis was done using ImageJ (NIH) / ImmunoRatio plugin, as described in the methods section. The sections were immunostained for expression of MGMT, p53, p21 and ki-67. Tumors from mice treated with BG +/- TMZ had a significant decrease in the expression of MGMT and ki-67. p53 expression was not significantly altered in these treatment groups. Expression of p21 was significantly increased in all treatment groups compared to controls (A). BG+/-TMZ also decreased phospho-proteins CDC2, TOP2A, AURKB, KIF20A and CDC20 expression in breast tumors. Temozolomide alone decreased phospho-TOP2A and phospho-AURKB expression in the breast tumors (B) correlating with the *in-vitro* expression results (B).

Tumors harvested from the four groups studied were processed for routine histological and IHC analysis. The BG and BG + TMZ combination treatment mediated, *in vivo*, correlative effects on MGMT, p53 and p21 and ki-67 were studied by IHC analysis and quantified using the ImmunoRatio plugin as described in the methods section. MGMT expression in tumors from mice treated with BG alone was decreased by 80% and by 90% by the BG+TMZ combination when compared to control. Benzylguanine decreased ki-67 expression by 95%. BG+TMZ combination decreased Ki-67 by 99%, when compared to control (Figure 7A). TMZ alone decreased ki-67 expression by 80%. p53 expression was not significantly altered across groups (Figure 7A). In sharp contrast, p21 expression was significantly increased in tumors from mice treated with BG alone or combination BG+TMZ (Figure 7A).

### BG induced MGMT inhibition leads to inhibition of CDC2, AURKB, TOP2A, KIF20A and CDC20 phosphorylation in breast cancer *in vivo*

*In vivo*, we have found that BG decreased expression of p-CDC2 (by > 80%), p-AURKB (by > 80%), p-TOP2A (by > 60%), and p-CDC20 (by > 60%) when compared to control. Further, when compared to control, BG+MGMT combination further decreased expression of p-CDC2 (99%), p-AURKB (99%), p-TOP2A (90%), and p-CDC20 (80%). Single agent TMZ had a limited inhibitory effect, when compared to control, on p-CDC2 (20%) and p-CDC20 (20%) and had a more significant inhibitory effect on p-AURKB (85%) and p-TOP2A (60%), (Figure 7B).

## DISCUSSION

MGMT expression level is very low in normal breast tissue and remains unchanged in normal tissue patient samples surrounding benign or malignant breast disease [1]. Increased MGMT expression is a hallmark of the tumor phenotype [1, 41] and directly correlates to the degree of local and distant tumor invasiveness [41]. In breast cancer, MGMT has been reported to control ER expression and function [1, 3, 4]. Increased and worsening metastatic burden and development of hormone therapy resistance in ER positive breast cancer correlates with increasing MGMT levels [4]. MGMT and ER expression and function correlation makes MGMT inhibition a possible option for reversal of hormonal therapy resistance [4].

Central to MGMT roles, beyond DNA damage repair, has been integration of DNA repair related signals with cell cycle progression and replication [8, 9].

In this study we show a correlation between MGMT expression and a variety of clinically relevant cell cycle regulators already known to play a role in breast oncogenesis.

BG induced MGMT protein inhibition correlated to decreased expression of CDC2, TOP2A, AURKB, KIF20A, CDC20, cyclin A2, B2, D1, survivin and ERα (Figure 1A and Supplementary Figure 4).

Further suppression of MGMT transcription via siRNA MGMT led to decreased expression in CDC2, TOP2A, AURKB, KIF20A, Cyclin B2, Cyclin A2 and survivin (Figure 2A), all associated with decreased transcription (Figure 3A–3E) and decreased expression of p-CDC2, p-TOP2A, p-AURKB, p-KIF20A, p-CDC20 (Figure 2B).

BG induced MGMT inhibition in our animal model was associated with decreased p-TOP2A, p-AURKB, p-CDC2, p-CDC20 expression (Figure 7B). MGMT inhibition in our model led to significant decrease in the multiple cell cycle regulatory targets tested suggesting that MGMT inhibition could be considered as an adjunct to distant inhibition of these multiple targets in the treatment of breast cancer.

Further MGMT inhibition could also have a significant clinical impact as an adjunct to chemotherapy by potentially enhancing activity of combination regimens including alkylator(s) (cytoxan – CMF; ifosfamide, carboplatin – ICE), anthracyclines or taxanes with potentially more significant impact when using regimens combining an alkylator with an anthracycline, when MGMT inhibition is also associated with TOP2A inhibition (AC, FAC/CAF, FEC) or an anthracycline with a taxene (AT) when MGMT inhibition is associated with both TOP2A and KIF20A inhibition.

In this study we also show that BG induced MGMT inhibition sensitizes ER positive MCF7 breast cancer cells to TMZ (Figure 5B). TMZ inhibits, in a dose dependent manner, ER positive breast cancer cell growth (Figure 1A). BG+/-TMZ inhibits MGMT activity (Figure 5C). Further, the BG induced MGMT inhibitory effect enhancing Temozolomide cell viability inhibitory effect extends to a variety of breast cancer cells, irrespective of presence or absence of 17-β estradiol (E2) (Supplementary Figure 2 and 3). TMZ is a weak alkylator taking 4-6 days to inhibit MGMT in breast cancer cells (Figure 4A). Inhibition of MGMT by TMZ is associated with induction of cytochrome c and p21 expressions in breast cancer cells (Figure 4B). BG inhibition of MGMT decreases CDC2, AURKB, TOP2A, KIF20A and CDC20 transcription (Figure 3A–3E). BG induced MGMT inhibition induces p21 transcription (Figure 3F). In addition, BG+/-TMZ significantly decreased p-CDC2, p-AURKB, p-TOP2A, p-KIF20A and p-CDC20 expression in breast cancer cells (Figure 1B). Temozolomide alone decreases p-AURKB and p-TOP2A expression in breast cancer cells compared to controls (Figure 1B). Temozolomide in combination with whole brain radiation did not significantly improve local control and survival in patients with brain metastases due to breast cancer [20] underscoring the role of MGMT over expression in metastatic breast cancer. As MGMT over expression is the most important predictor for temozolomide resistance [42] this study confirms in a preclinical breast cancer model that MGMT inhibition could increase sensitivity to temozolomide in breast cancer suggesting a basis for reconsideration of Temozolomide use in combination with an MGMT inhibitor in patients with brain metastatic disease. Others have already reported in an ER positive breast cancer xenograft model the benefit of lomeguatrib (an oral MGMT inhibitor) in restoring temozolomide sensitivity [43].

Our present and prior data [4] suggest a role for MGMT inhibition in the treatment of breast cancer which could enhance multiple breast cancer treatment options including (alkylator, anthracycline or taxane based) chemotherapy, restore sensitivity to hormonal therapy [4] or potentially delay hormonal therapy resistance. The role of MGMT inhibition in ER positive breast cancer could be further expanded. We have also reported [44] that inhibition of MGMT in an *in vitro* breast cancer model is associated with CDK4 inhibition and enhances palbociclib and abemaciclib effect. Further, mTOR inhibition is central to some of the combination therapies used in clinical practice for hormonal refractory disease. As mTOR inhibition has been reported to be associated with increased levels of MGMT protein via post-translational mechanisms [45, 46], one could argue that a combination of mTOR inhibition and MGMT inhibition/depletion would work better in restoring/expanding hormonal sensitivity.

MGMT inhibition via BG has lately been avoided in clinical trials as it was associated with significant hematologic toxicity when used with standard alkylator doses. There currently are two other clinically viable options for MGMT inhibition – lomeguatrib [47] (which is not approved for use in the US) and disulfiram (especially when combined with copper) [48, 49]. Lomeguatrib works the same way as BG, both O6-meG pseudo-substrates leading to MGMT protein inactivation [47] and, as both could lead to hematologic toxicity, one could argue a need for a dose reduction in the concomitant alkylator (or other chemotherapy agent) in order to mitigate hematologic toxicity. Disulfiram has received FDA approval for the treatment of alcoholism in 1951 and has been known to have anticancer effects for more than 50 years. Disulfiram anticancer activity has been under renewed scrutiny from a mechanistic stand point as disulfiram shows multiple anticancer effects including MGMT inhibition and MGMT depletion [48]. Our study suggests that MGMT inhibition should be further evaluated in preclinical studies and clinical trials as potential enhancer of chemotherapy and as an option for restoration of hormonal sensitivity.

## MATERIALS AND METHODS

### Cell culture

Breast cancer cell lines (MCF7 and ZR-75 – both ER and MGMT positive, ZR-75-30, HCC 1428 and MDA MB 468 – MGMT positive and ER weakly positive/negative) purchased from the American Tissue Culture Collection (ATCC; Manassas, VA) were grown in DMEM (MCF-7, T-47D, MDA MB 468) and RPMI 1640 (ZR-75-1, ZR-75-30, HCC 1428) medium. All the media were supplemented with 10% fetal bovine serum and 1% penicillin-streptomycin (GIBCO, Invitrogen Corporation, NY). Adherent monolayer cultures were maintained at 37°C containing 5% CO_2_.

### Antibodies and drugs

p53 (#sc-126), normal mouse IgG and HRP (#sc-2025; #sc-2748) and ERα (#sc-543) were purchased from Santa Cruz Biotechnology, Santa Cruz, CA. MGMT (#MAB16200), ki-67 (#MAB4190), AURKB (#MAB 04-1036) and TOP2A (#MAB 4197MI) antibodies were purchased from Chemicon-Millipore, Billerica, MA. CDC2 (#9112S), phospho-CDC2 (Try15) (#9111S), phospho-CDC2 (Tyr14) (#2543S), CDC20 (#4823S), phospho-CDC20 (ser51) (#8038), p-AURKB (#2914S), Cyclin A2 (#4656S), Cyclin D1(#2922), survivin (#2803), p21 (#2946), cleavedPARP (#9546), cytochrome C (#4272) and PUMA (#4976) were purchased from Cell Signaling, Beverly, MA. Phospho-TOP2A (p-s1106) (#ab75765), KIF20A (#ab70791), phospho-KIF20A (p-s528) (#ab63547) and Cyclin B2 (#ab82287) were purchased from Abcam, Cambridge, MA. β-Actin (#A2066) antibody was purchased from Sigma-Aldrich, St. Louis, MO. O^6^ benzyl guanine (BG; MGMT Blocker) was purchased from Sigma-Aldrich (St. Louis, MO). Temozolomide was obtained from Aurora Saint Luke’s Pharmacy. BG and TMZ were dissolved in DMSO and subsequent dilutions were made with tissue culture media. Equal amounts of DMSO were used in tissue culture media for untreated controls.

### Western blotting

MCF7 cells were plated overnight (1 × 10^6^) in 10 cm^2^ petri dishes and were subsequently treated with TMZ (IC50 for MCF7 cells - 644μM) containing media at every two days. Further, cells were harvested after 2, 4 and 6 days, washed twice with 1X PBS and lysates were prepared using 1X Laemmli buffer (Bio-Rad), total proteins were estimated using BCA Protein Assay Kit (Pierce, Rockford, IL) and western blot analysis was performed. In another set of experiments MCF7 cells (1 × 10^6^) were plated overnight in 10 cm^2^ petri dishes and were subsequently treated at every 2 days with different concentrations of TMZ (257μM-772μM) and cells were harvested 4 days post-treatment. In another set of experiments, MCF7 cells (1 × 10^6^) were plated overnight in 10 cm^2^ petri dishes and were subsequently treated at every 2 days with BG+TMZ (50 μM + 644 μM) before harvest 4 days post-treatment. In another set of experiments, MCF7 cells (1 × 10^6^) were plated for 24 hours and further transfected with NT (non-target) siRNA (100 nM) and MGMT siRNA (100 nM) for 72 hours before western blot analysis. Cells were harvested and washed twice with 1X PBS and lysates were made using 1X Laemmli buffer before proteins were separated on 10% SDS-PAGE gels, transferred to nitrocellulose membranes followed by standard procedure [50–52]. In a supplementary set of experiments breast cancer cells (MCF-7, T-47D, ZR-75-1, ZR-75-30, HCC 1428 and MDA MB 468) were plated overnight (1 × 10^6^) in 10 cm^2^ petri dishes and proteins were isolated and western blot was performed to characterize ERα and MGMT expression levels. In another set of supplementary experiments MCF-7 and ZR-75-1 cells were plated overnight and further treated with single agents (TMZ - 64μM – lowest concentration noted to have a growth inhibitory effect, BG - 50 μM - lowest concentration noted to have a growth inhibitory effect, E2 – 10 nM) or combinations (TMZ+BG, TMZ+E2, BG+E2 and TMZ+BG+E2) every other day before harvest at 4 days and proteins were isolated and western blot was performed.

### Cell viability assay

MCF7 and ZR-75 cells (2 × 10^3^/well) were plated overnight in 96-well plates (Lonza, Rockland, ME) and subsequently treated with different concentrations of TMZ (64μM – 1mM) at every 2 days and harvested 4 days post-treatment. Cell viability was determined by measuring the ATP amount - using the Cell Titer-Glo Luminescent Cell Viability Assay (Promega, Madison, WI). The detection of luminescence (RLU) was measured by Optima Fluor Star Luminometer (BMG Lab Tech, Cary, NC). In a supplementary set of experiments breast cancer cellsMCF7, ZR-75-1, T-47-D, ZR-75-30, HCC -1428 and MDA MB 468 were treated with single agents (at low concentration TMZ 64μM and BG 50μM) in presence or absence of 17β – estradiol (E2) (10nM) or combination therapy (TMZ+BG+/-E2) and cell viability was measured.

### Quantitative real-time PCR

For qRT-PCR, MCF7 breast cancer cells (1 × 10^6^) were plated overnight and the next day were treated with TMZ (644 μM) +/-BG (50 μM) at every 2 days before harvest 2 and 4 days post-treatment. Cells were lysed using Trizol reagent (Invitrogen, CA). Total RNA was isolated using Qiagen columns (Qiagen, Valencia, CA) and reverse transcribed using SuperScript ™ First Strand Synthesis System for RT-PCR (Cat. No. 12371-019). Quantitative real-time PCR (qRT-PCR) was performed using ABI 7300 sequence detection system (Applied Biosystems, Carlsbad, CA). TaqMan gene expression assays for CDC2 (Hs00938777_m1^*^); CDC20 (Hs00426680_mH^*^); AURKB (Hs00177782_m1); TOP2A (Hs00172214_m1); KIF20A (Hs00993573); p21 (Hs00355782_m1) and β-actin (4333762F) were purchased from Applied Biosystems, Carlsbad CA. The relative mRNA levels of p53 and p21 were calculated using the ∆∆ Ct method, with the β-actin mRNA as an endogenous control.

### MGMT activity assay

Exponentially growing MCF7 cells were treated with BG (50μM) and/or TMZ (644μM). After 48hrs media with or without drugs was changed and cells were harvested after 96h. Cells were trypsinized and washed with Tris-buffered saline (TBS; pH 8.0). Cell-free extracts were prepared by sonication in MGMT assay buffer containing 40 mM Tris-HCl (pH 8.0), 5% glycerol, 1 mM EDTA, 0.5 mM DTT, 50 μM spermidine and 1 mM PMSF followed by centrifugation at 10,000 × g for 10 min. The substrate DNA enriched in O^6^-methylguanine was prepared by using [methyl ^3^H]-methylnitrosourea (60 Ci/mmol; GE Healthcare, Pittsburgh, PA) according to Myrnes et al. [53]. MGMT activity was determined by quantitating the transfer of the ^3^H-labeled methyl group from the O^6^ position of guanine in the DNA to the MGMT protein [54]. Briefly, cell extracts (50–200 μg of protein) were supplemented with 2μg of DNA substrate (∼10,000 cpm) and incubated at 37°C for 30 min, after which the DNA was hydrolyzed in trichloroacetic acid at 80°C for 30 min. The protein precipitates were collected on glass fiber filters, washed with 5% TCA and ethanol, solubilized, and the radioactivity was counted.

### Orthotopic injection of breast cancer cells in athymic nude mice

Female ovarectomized athymic nude mice were purchased from Harlan (Indianapolis, IN). The mice were housed and maintained in specific pathogen-free conditions. The mice were studied when they were 6 to 8 weeks of age, in accordance with institutional guidelines as stipulated by our IACUC. To develop tumors, MCF7 luc cells (MCF7 cells were tagged with luciferase expression vector to monitor tumor growth by Kodak Imaging System, Rochester, NY) were harvested from subconfluent cultures by brief exposure to 0.05% trypsin and 0.02% EDTA. Trypsinization was stopped with a medium containing 10% fetal bovine serum as the cells were washed once in a serum-free medium and re-suspended in HBSS. Only suspensions consisting of single cells with >90% viability were used for injections. A 1.7 mg time release β-estradiol pellet (Innovative Research of America, Sarasota, FL) was implanted over the right shoulder of each mouse and 24 hrs later MCF7 luc (7 × 10^6^) cells were injected into the mammary fat pad.

### Therapy of established human MCF7 breast cancer cells growing in the mammary fat pad of nude mice

In previous studies in our laboratory, we determined that BG at 10mg/kg and TMZ at 100mg/kg were well tolerated by mice as indicated by preservation of body weight [4]. In subsequent experiments BG was administered daily at 10mg/kg by i.p. injection and TMZ was administered twice weekly at 100mg/kg by i.p. injection. Ten days after injection of MCF-7 cells into the mammary fat pad, the mice were randomized into four groups (n/group = 10) as follows: (a) daily (Mon-Fri) i.p injections of 1X PBS in control groups, (b) daily (Mon-Fri) i.p. injections of benzyl guanine (10mg/kg), (c) twice weekly (Mon and Thu) i.p. injections of TMZ (100mg/kg), (d) combination therapy BG (10mg/kg, five days/week) and TMZ (100mg/kg, twice/week). Treatments were continued for 8 weeks when the mice were euthanized and subjected to necropsy. Tumor volumes were calculated using the following formula: (length)^2^ × (width) / 2. Body weight was not changed among all treatment groups as compared with control mice. No visible liver metastases were present (enumerated with the aid of a dissecting microscope) in any of the treatment groups. All slides were reviewed by a board certified pathologist.

### Histological studies

For immunohistochemistry and histologic staining, paraffin embedded tissues were used to detect protein expression of MGMT, p53, p21, ki-67, p-CDC2, p-AURKB, p-TOP2A and p-CDC20. Sections (4 – 6 μm thick) were mounted on positively charged superfrost slides (Fischer Scientific, Co., Houston, TX) and dried overnight. Sections were deparaffinized in xylene and subsequently treated with a graded series of alcohol (100%, 95%, and 80% ethanol [vol/vol] in deionized H_2_O) and re-hydrated in deionized H_2_O and PBS (pH 7.5). Antigen retrieval for MGMT, p53, p21 and ki-67 was achieved by placing slides in 100°C 0.1M citrate buffer (pH 6.0) for 10 minutes, followed by 30 minutes of bench top cooling. Slides were washed with PBS that contained 0.1% triton and 0.1% BSA. Antigen retrieval for p-CDC2, p-CDC20, p-AURKB, and p-TOP2A was achieved by placing slides in 100°C 1mM EDTA (pH 8.0) for 60 minutes, and then washed with PBS. Endogenous peroxidase was blocked with 3% hydrogen peroxide in PBS, while nonspecific binding was blocked with 10% normal horse serum (10% goat serum for phosphorylated antibodies in 1% BSA in PBS) and 2% BSA in PBS. The slides were incubated at 4°C overnight in a moist chamber with one of the following: 1) monoclonal mouse anti-MGMT antibody (Invitrogen, Carlsbad, CA; #35-700; 10 μg/ml) or 2) monoclonal mouse anti-p53 antibody (Santa Cruz, Santa Cruz, CA; #sc126; 1:50 dilution) or 3) mouse anti ki-67 antibody (Millipore, Billerica, MA; #MAB 4190; 1:50 dilution) or 4) monoclonal mouse anti-p21 (Cell Signaling, Beverly, MA; #2946; 1:100 dilution) or 5) monoclonal rabbit p-TOP2A (phosphoS1106) antibody (Abcam, Cambridge, MA; ab75765; 1:100 dilution) or 6) monoclonal rabbit p-ARUKB (Thr232) antibody (Cell Signaling, Beverly, MA; #2914; 1:50 dilution) or 7) monoclonal rabbit p-CDC2 (Tyr15) (10A11) antibody (Cell Signaling, Beverly, MA; #4539; 1:100 dilution) or 8) polyclonal rabbit p-CDC20 (Ser51) antibody (Cell Signaling, Beverly, MA; #8038;1:100 dilution). Slides were washed with PBS that contained 0.1% triton and 0.1% BSA. After one hour incubation at room temperature with a biotinylated horse anti-mouse IgG/streptavidin complex (VWR, PA; #PI32052) or goat anti-rabbit IgG/streptavidin complex (VWR, PA; #8010), a positive reaction was visualized by incubating the slides with stable 3,3′-diaminobenzidine (Invitrogen Corporation, Carlsbad, CA) for 10-15 minutes. Counter-staining was achieved by rinsing the sections with two changes of tap water, placing them in Gill’s filtered hematoxylin (EMD Chemicals, Philadelphia, PA) for 10 minutes, followed by successive dips into tap water, acid alcohol (EMD Chemicals, Philadelphia, PA), tap water, lithium carbonate (EMD Chemicals, Philadelphia, PA) and tap water. All slides were mounted with Crystal Mount (Fischer Scientific, Co., Houston, TX). Control samples exposed to secondary antibody alone showed no specific staining. The images were analyzed by ImageJ (NIH) and MGMT, p53, p21, ki-67 and CDC2, TOP2A, AURKB and CDC20 expressions were quantified using the ImmunoRatio plugin [55]. Results were reported as mean ± SD as for each condition 5 contiguous slides were generated and analyzed. All slides were reviewed by a board certified pathologist.

### Statistical analysis

Experiments were performed in triplicates and data presented as mean ± SD. Statistical analysis was done using Student’s *t* test, assuming equal variance, and each *p* value was calculated based on two-tailed test. A *p* value of <0.05 was considered statistically significant. *P* values are reported using a star system as follows: ^*^p<0.05; ^**^p<0.005 and ^***^p<0.0005.

## SUPPLEMENTARY MATERIALS FIGURES



## Abbreviations

MGMT: O^6^ methylguanine DNA methyltransferase
BG: O^6^ Benzylguanine
TMZ: Temozolomide
qRT-PCR: Quantitative Reverse Transcriptase Polymerase Chain Reaction (real-time PCR)
