# Supplementary material for: MGMT inhibition in ER positive breast cancer leads to CDC2, TOP2A, AURKB, CDC20, KIF20A, Cyclin A2, Cyclin B2, Cyclin D1, ERα and Survivin inhibition and enhances response to temozolomide
Source: Oncotarget. 2018 Jul 3;9(51):29727–42. doi: 10.18632/oncotarget.25696 (PMC6049872; doi:10.18632/oncotarget.25696)
Supplement: Supplementary file 1 [file oncotarget-09-29727-s001.pdf]

MGMT inhibition in ER positive breast cancer leads to CDC2, TOP2A, AURKB, CDC20, KIF20A, Cyclin A2, Cyclin B2, Cyclin D1, ERα and Survivin inhibition and enhances response to temozolomide

SUPPLEMENTARY MATERIALS

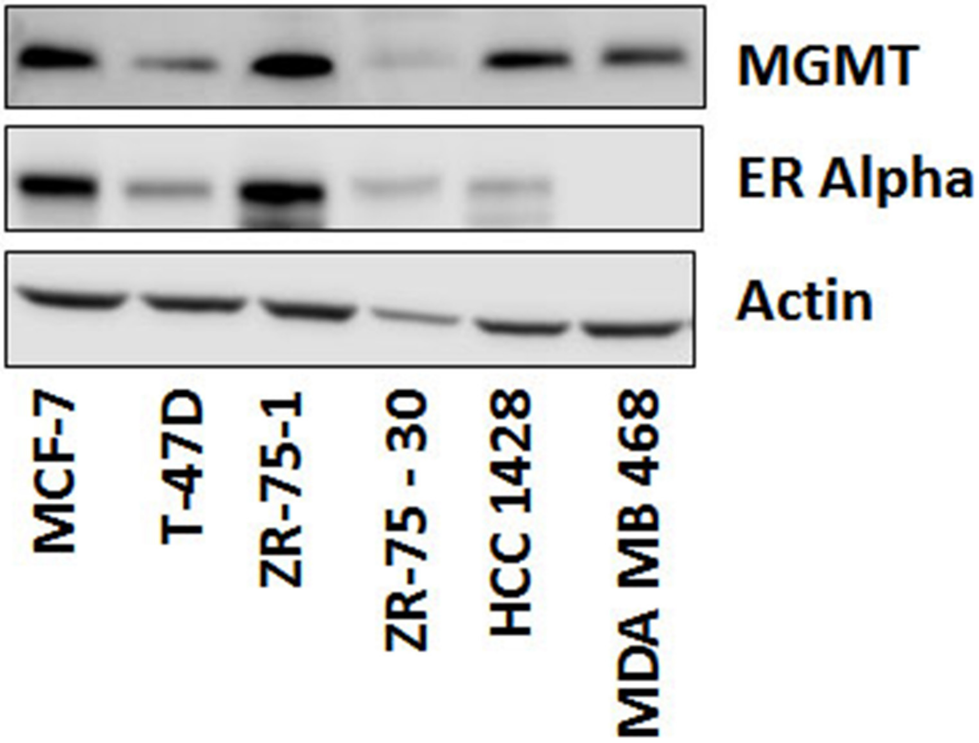

Supplementary Figure 1: ERα and MGMT expression in breast cancer cells. Breast cancer cells plated overnight were used to isolate proteins and western blot was performed to investigate MGMT and ERα expression. MGMT and ERα were present in all these cells except MDA MB 468.

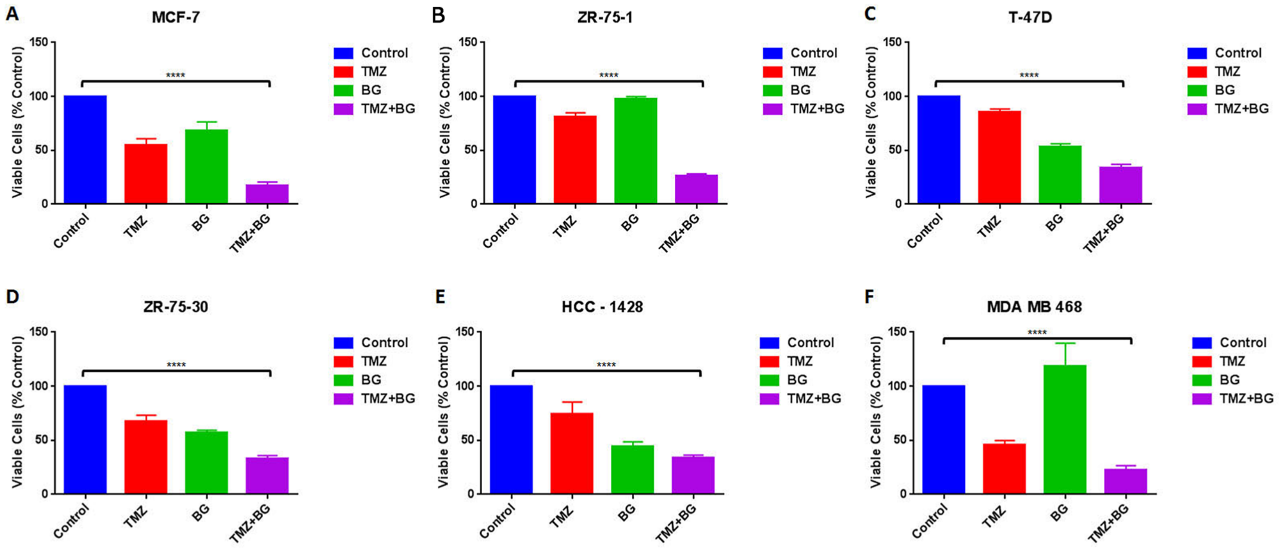

Supplementary Figure 2: BG+/-TMZ therapy decreases breast cancer cells viability. Human breast cancer cells MCF7 **(A)**, ZR-75-1 **(B)**, T-47-D **(C)**, ZR-75-30 **(D)**, HCC-1428 **(E)** and MDA MB 468 **(F)** cells were plated overnight and treated with TMZ at low concentration (64µM) and BG at low concentration (50µM) every other day before harvest at 4 days and ATP assays were performed. Combination therapy significantly decreased cell viability of all these cells compared to single agents and untreated controls.

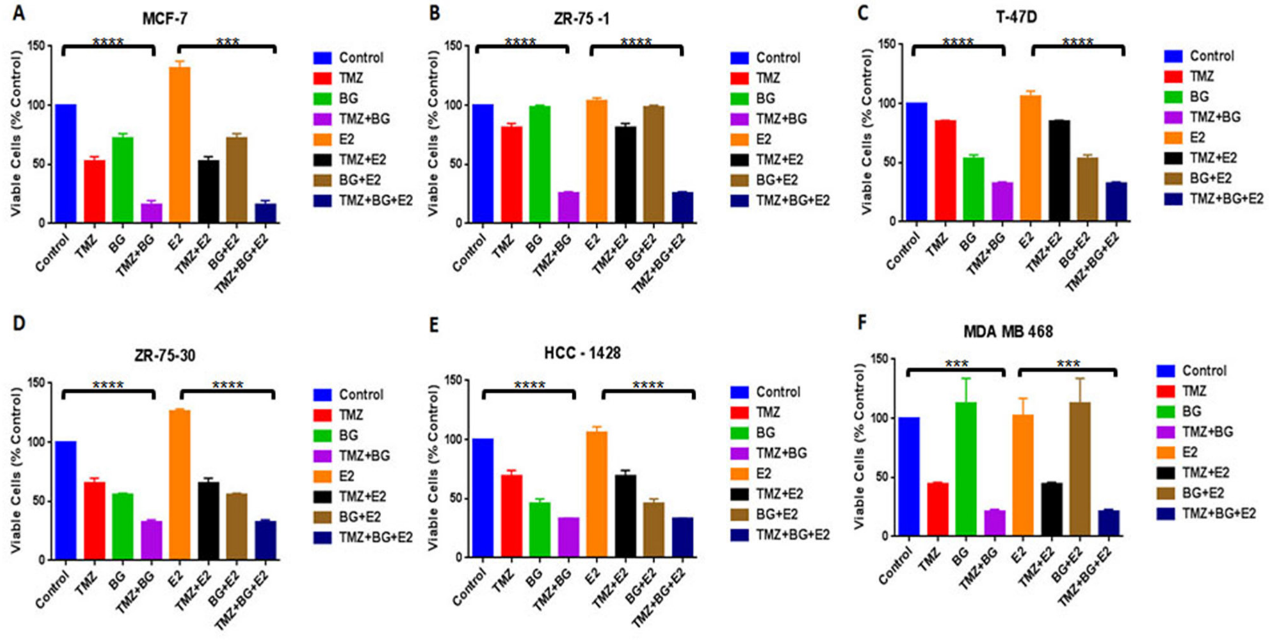

Supplementary Figure 3: Presence of 17-β estradiol (E2) does not alter *in vitro* BG+/-TMZ inhibitory effect. Human breast cancer cells MCF7 **(A)**, ZR-75-1 **(B)**, T-47-D **(C)**, ZR-75-30 **(D)**, HCC-1428 **(E)** and MDA MB 468 **(F)** cells were plated overnight and further treated with TMZ (64µM) and BG (50µM) every other day in presence or absence of 17β – estradiol (E2 10nM) before harvest at 4 days and ATP assays were performed. Combination therapy significantly decreased cell viability of all these cells in presence or absence of E2 compared to single agents and untreated controls. In addition, E2 mediated growth stimulatory effect did not alter/cancel the growth inhibitory effect of combination therapy.

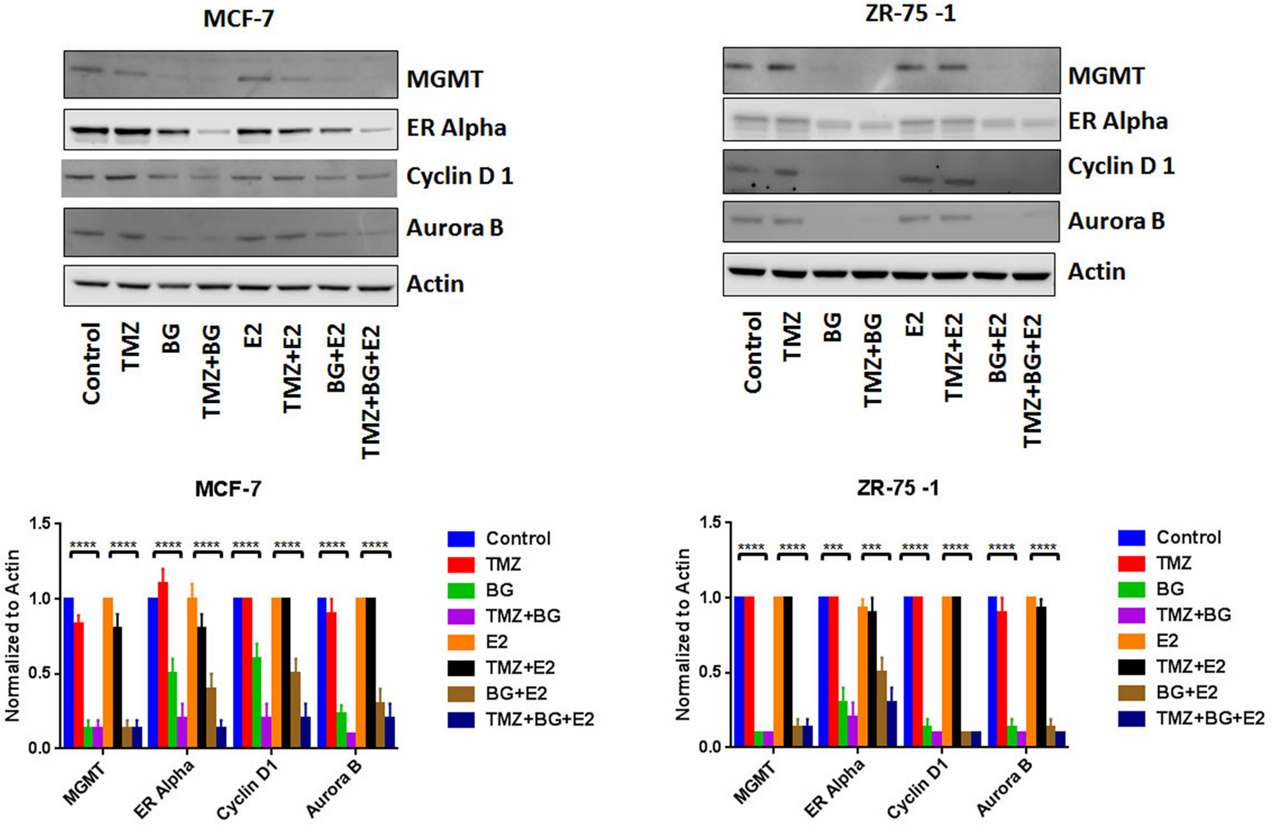

Supplementary Figure 4: Presence of 17-β estradiol (E2) does not alter BG+/-TMZ therapeutic effect on MGMT and ERα downstream targets (Cyclin D1 and ARUKB). Human breast cancer cells plated overnight (MCF-7 and ZR-75-1) were further treated with single agents (TMZ - 64μM, BG - 50 μM, E2 – 10 nM) or combinations (TMZ+BG, TMZ+E2, BG+E2 and TMZ+BG+E2) every other day before harvest at 4 days and proteins were isolated and western blot was performed. MGMT, ERα, Cyclin D1 and Aurora Kinase B expressions were evaluated. BG and TMZ+BG significantly decreased all these protein expressions irrespective of presence or absence of E2.
